# Supplementary material for: Metabolomic Profiling of End-Stage Heart Failure Secondary to Chronic Chagas Cardiomyopathy
Source: Int J Mol Sci. 2022 Sep 9;23(18):10456. doi: 10.3390/ijms231810456 (PMC9499603; doi:10.3390/ijms231810456)
Supplement: Supplementary file 1 [file ijms-23-10456-s001.zip › Supplementary_Figure.pdf]

Metabolomic Profiling of End-Stage Heart Failure Secondary to Chronic Chagas Cardiomyopathy.

Supplementary File

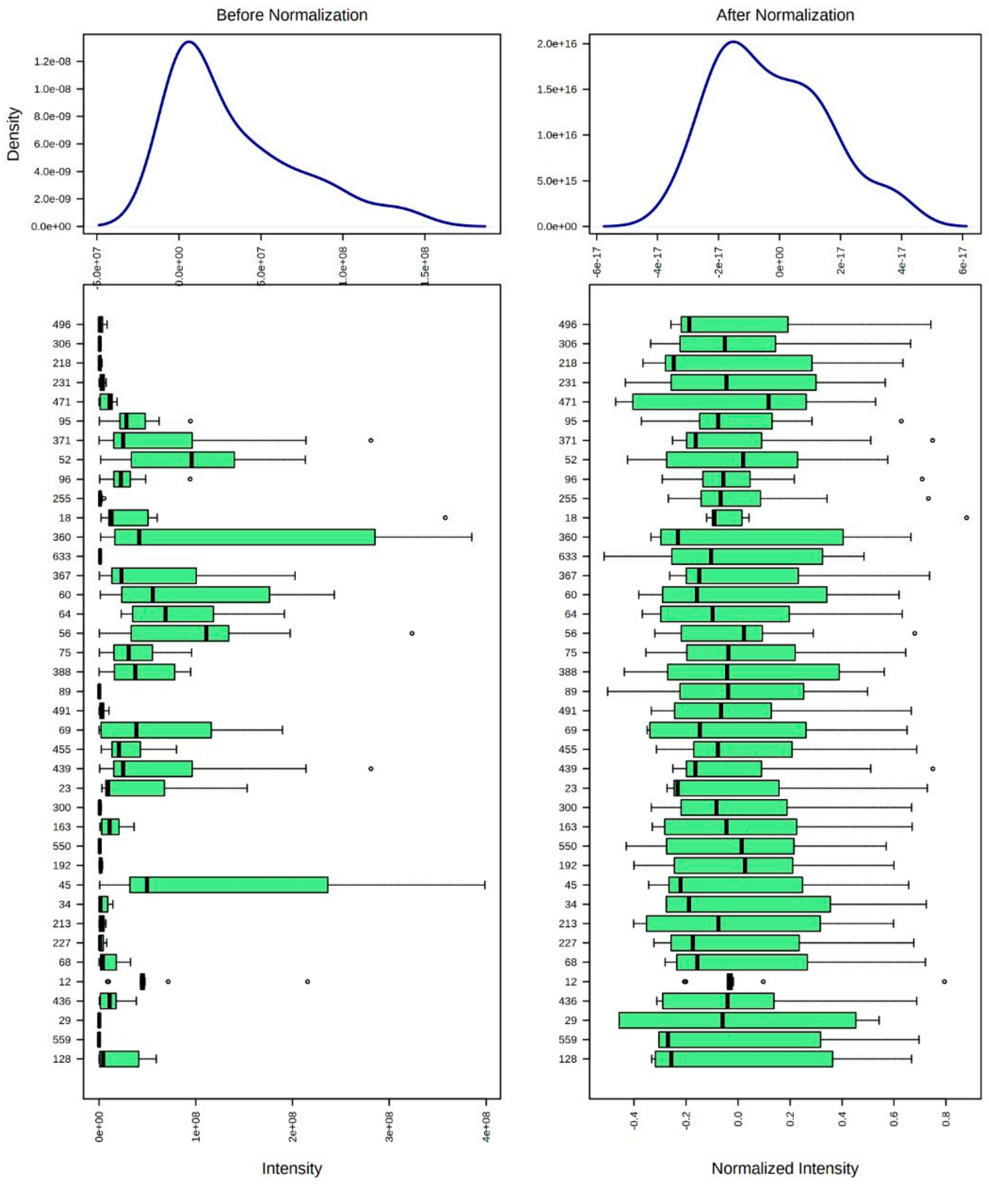

Supplementary Figure S1. Changes in the distribution of the data before and after the normalization process.
